# The Mitochondrial PHB Complex Determines Lipid Composition and Interacts With the Endoplasmic Reticulum to Regulate Ageing

**DOI:** 10.3389/fphys.2021.696275

**Published:** 2021-07-01

**Authors:** Artur B. Lourenço, María Jesús Rodríguez-Palero, Mary K. Doherty, David Cabrerizo Granados, Blanca Hernando-Rodríguez, Joaquín J. Salas, Mónica Venegas-Calerón, Phillip D. Whitfield, Marta Artal-Sanz

**Affiliations:** ^1^Andalusian Centre for Developmental Biology (CABD), CSIC-Universidad Pablo de Olavide-Junta de Andalucía, Seville, Spain; ^2^Department of Molecular Biology and Biochemical Engineering, Universidad Pablo de Olavide, Seville, Spain; ^3^Division of Biomedical Science, University of the Highlands and Islands, Inverness, United Kingdom; ^4^Instituto de la Grasa (CSIC), Universidad Pablo de Olavide, Seville, Spain

**Keywords:** mitochondria, prohibitin (PHB), insulin, ageing, lipidomics, yolk, UPR^ER^, lipid droplet

## Abstract

Metabolic disorders are frequently associated with physiological changes that occur during ageing. The mitochondrial prohibitin complex (PHB) is an evolutionary conserved context-dependent modulator of longevity, which has been linked to alterations in lipid metabolism but which biochemical function remains elusive. In this work we aimed at elucidating the molecular mechanism by which depletion of mitochondrial PHB shortens the lifespan of wild type animals while it extends that of insulin signaling receptor (*daf-2*) mutants. A liquid chromatography coupled with mass spectrometry approach was used to characterize the worm lipidome of wild type and insulin deficient animals upon PHB depletion. Toward a mechanistic interpretation of the insights coming from this analysis, we used a combination of biochemical, microscopic, and lifespan analyses. We show that PHB depletion perturbed glycerophospholipids and glycerolipids pools differently in short- versus long-lived animals. Interestingly, PHB depletion in otherwise wild type animals induced the endoplasmic reticulum (ER) unfolded protein response (UPR), which was mitigated in *daf-2* mutants. Moreover, depletion of DNJ-21, which functionally interacts with PHB in mitochondria, mimicked the effect of PHB deficiency on the UPR^ER^ and on the lifespan of wild type and insulin signaling deficient mutants. Our work shows that PHB differentially modulates lipid metabolism depending on the worm’s metabolic status and provides evidences for a new link between PHB and ER homeostasis in ageing regulation.

## Introduction

Ageing and the increasing prevalence of age-related pathologies are a major concern worldwide. Nutrient-sensing pathways and mitochondria maintain metabolic homeostasis, being metabolic alterations a hallmark of ageing (Toth and Tchernof, 2000; Niccoli and Partridge, 2012). *Caenorhabditis elegans* has contributed enormously to our understanding of the ageing process as many metabolic genes are conserved between worms and humans (Kenyon, 2010; Shaye and Greenwald, 2011; Lemieux and Ashrafi, 2015). The worm intestine is perhaps its most metabolically active organ, performing fat storage and liver-like functions (McGhee, 2013). Vitellogenesis, which leads to the formation of yolk particles (YPs), occurs in the intestine and has a major impact on lipid homeostasis. YPs, carrying lipids like triacylglycerol and different phospholipids, are mobilized to the gonad where they are taken up by developing oocytes (Kimble and Sharrock, 1983; Spieth and Blumenthal, 1985; Heine and Blumenthal, 1986; Sharrock et al., 1990; Grant and Hirsh, 1999; McGhee, 2013). In addition, lipid homeostasis within a cell involves the dynamic interaction between organelles like the endoplasmic reticulum (ER), lipid droplets (LD) and mitochondria (Gami and Wolkow, 2006; Nguyen et al., 2017; Sugiura et al., 2017; Diogo et al., 2018).

The mitochondrial prohibitin complex (PHB) contributes to mitochondrial biogenesis, structure, functionality, and degradation (Nijtmans et al., 2002; Artal-Sanz and Tavernarakis, 2009a; Hernando-Rodriguez and Artal-Sanz, 2018). PHB is composed of 12 to 16 PHB-1/-2 heterodimers assembled into a ring-like structure in the mitochondrial inner membrane (Back et al., 2002; Tatsuta et al., 2014). Although its exact molecular function is still unknown, several phenotypes have been ascribed to PHB (Nijtmans et al., 2000; Osman et al., 2009; Richter-Dennerlein et al., 2014). Particularly intriguing is its evolutionarily conserved effect on lifespan depending on the organism’s metabolic status (Artal-Sanz and Tavernarakis, 2009b, 2010; Schleit et al., 2013). Prohibitin complex depletion shortens the lifespan of otherwise wild type nematodes while extending the lifespan of a variety of metabolically compromised mutant genetic backgrounds, including the long-lived *daf-2(e1370)* mutant (Artal-Sanz and Tavernarakis, 2009b). DAF-2, the ortholog of the insulin/insulin-like growth factor (insulin/IGF) receptor, modulates the activity of the insulin/IGF-1 signaling (IIS) pathway, through the transcription factor DAF-16, which is required to regulate ageing, metabolism and reproductive growth (Kenyon et al., 1993; Pierce et al., 2001; Li et al., 2003; McElwee et al., 2003). Indeed, *daf-2* mutants are characterized by metabolic rewiring and increased triacylglycerol content stored in large LDs (Murphy et al., 2003; Halaschek-Wiener et al., 2005; Ruzanov et al., 2007; Depuydt et al., 2014; Narayan et al., 2016; Gao et al., 2018). Importantly, the expression of the ATGL-1 lipase, a LD-associated protein (Lee et al., 2014), is regulated by the DAF-2/DAF-16 axis and required for the increase longevity of *daf-2* mutants (Zaarur et al., 2019).

Recently, in mammalian cells, PHB has been proposed to cooperate with the mitochondrial cochaperone DNAJC19, for which DNJ-21 is the worm homolog, in the remodeling of mitochondrial membrane phospholipids (Richter-Dennerlein et al., 2014). In *C. elegans*, PHB depletion modulates differently the whole worm fatty acid composition of wild type and *daf-2* mutants (Lourenco et al., 2015). Still, the broader impact of PHB in the whole lipidome has never been assessed. Herein, we found that PHB modulates triacylglycerol and phospholipids pools at the young adult (YA) stage and during chronological ageing in a genetic background-dependent manner. Moreover, we provide, for the first time, data supporting a cross-talk between PHB and the ER in the regulation of lipid metabolism and ageing.

## Materials and Methods

### Strains and Worm Culture

Nematodes were cultured and maintained according to standard methods. The *C. elegans* strains used in this study were: N2, wild type Bristol isolate, CB1370: *daf-2(e1370)III*, RT130 *unc-199(ed3)*; *pwIs23[Pvit-2:vit-2:GFP;unc-199(*+)*]*, MRS416: *daf-2(e1370)III*;*pwIs23[Pvit-2:vit-2:GFP;unc-199(*+)*]* (this study), BC12843: *dpy-5(e907)I*;*sIs11286[rCesK07H8.6:GFP* + *pCeh361]*, MRS402: *daf-2(e1370)III*;*sIs11286[rCesK07H8.6:GFP* + *pCeh361]* (this study), SJ4005: *zcIs4[hsp-4:GFP]*, MRS484: *daf-2(e1370)III*;*zcIs4[hsp-4:GFP]* (this study), MRS78: *phb-1(tm2751)I/hT2[bli-4(e937)qIs48(Pmyo-2:GFP)](I,III);zcIs4[hsp-4:GFP]V*, VS29: *hjSi56[vha-6p:3xFLAG:TEV:GFP:dgat-2:let-858 3’UTR]*, MRS269: *daf-2(e1370)III*;*hjSi56[vha-6p:3xFLAG:TEV:GFP:dgat-2:let-858 3’UTR]* (this study), VS20: *hjIs67[atgl-1p:atgl-1:gfp* + *mec-7:rfp]*, MRS 270: *daf-2(e1370) III;hjIs67 [atgl-1p:atgl-1:gfp* + *mec-7:rfp]* (this study) and VZ188: *vzEx64 [Pdnj-27:GFP]*. In all experiments, worms were grown at 20°C on NGM plates seeded with HT115 (DE3) *Escherichia coli* bacteria (deficient for RNase-E) harboring the appropriate RNAi plasmids (pL4440 for Control RNAi, pL4440 containing *phb-1* or *dnj-21* genomic fragment). Overnight cultures of each bacterial strain were used to inoculate liquid LB [carbenicillin (25 mg/l), tetracycline (15 mg/l) (Sigma)] and incubated until an OD_600__nm_ of 1.5 (37°C). Next, IPTG [1 mM (Sigma)] was added to the culture, incubated for 2 h and harvested by centrifugation (3,200 × *g*, 20 min, 4°C). Pellets were washed with S Basal and harvested again (4°C). Finally, pellets (30 g/l) were resuspended in S Medium containing carbenicillin (25 mg/l), IPTG (1 mM) and cholesterol (5 mg/l) (Sigma), and bacterial stocks were kept at most 4 days before use (4°C). Synchronous worm populations were generated by allowing gravid adults to lay eggs on seeded NGM plates. Tightly synchronized populations were obtained by allowing eggs, generated by hypochlorite treatment of gravid adults, to hatch and develop until L1 larval stage in S Basal during overnight incubation (20°C).

### LC-MS Analysis

Tightly synchronized populations (30,000 worms) were sampled at YA stage, washed [three times with S Basal, two with double distilled water, once with MS-grade water (Fluka); 20°C], harvested after each washing step by centrifugation (800 × *g*, 1 min, 20°C), and immediately snap-frozen in liquid nitrogen and kept in the freezer (<-80 °C). A protein assay was undertaken to ensure consistent sampling of the worm population prior to lipid analysis. Lipids were extracted according to the method of Folch (Folch et al., 1957). Lipid extracts were separated on a Hypersil Gold C18 (2.1 mm x 100 mm, 1.9 μm column) connected to a Accela UPLC and Exactive Orbitrap mass spectrometer (Thermo Fisher Scientific) equipped with a heated electrospray ionization (HESI) probe. In the chromatographic method, mobile phase A consisted of water with 10 mM ammonium formate and 0.1% formic acid, and mobile phase B consisted of 90:10 isopropanol/acetonitrile, with 10 mM ammonium formate and 0.1% formic acid. The starting condition was 65% A/35% B, then buffer B increased to 100% over 10 min and held at this composition for 7 min before re-equilibration at the starting condition for 4 min. All samples were analyzed in both positive and negative ionization mode and the scan range between m/z 120 and 2000. Data was processed using Progenesis CoMet software (Non-linear Dynamics, Newcastle, United Kingdom) and searched against LIPIDMAPS and HMDB for identification. Multivariate statistical analysis was performed with SIMCA 13 (Umetrics, Umea, Sweden).

### HPLC and TLC Analyses

Tightly synchronized populations were sampled (20,000 worms) at days 0, 6, and 10 of adulthood, washed like previously described and then worm pellets were immediately boiled in 2 ml of hot methanol (15 min). Next, 4 mL of chloroform were added and the pellet was homogenized using the Sonopuls HD270 (Bandelin). After 1 h of incubation at room temperature, phase separation was initiated by adding 1.4 ml of 0.88% KCl. 3.5 ml lipid-rich lower chloroform layer was collected and kept in the freezer (<-20°C). Lipid extracts were evaporated under nitrogen and the residue was dissolved in 5 ml of chloroform. The resulting solution was fractionated in a Lichrolut 0.5 g silica gel cartridge (Merck) using a vacuum manifold and equilibrated with 2 ml of chloroform. Subsequently, 15 ml of chloroform was used to elute neutral lipids. The column was then washed with 10 ml of methanol to recover the polar fraction. Neutral lipids were evaporated under nitrogen and dissolved in 3 ml of hexane. Polar lipids were evaporated under nitrogen and dissolved in 1.5 ml of hexane/2-propanol (3:2 v/v).

Quantification by HPLC was carried out in a Waters 2695 Module (Milford, MA) equipped with a Waters 2420 ELSD evaporative light scattering detector. Polar and neutral lipids were separated at 30°C using a Lichrospher 100 Diol 254-4 (5 μm) column or a normal phase Lichrocart 250-4 (5 μm) column (Merck) applying the methods described by Salas et al. (2006). For polar lipids, the column was equilibrated with 100% hexane/2-propanol/acetic acid/Trimethylamine (82:17:1:0.08 v/v/v/v). The sample was injected and a gradient from 0 to 40% of 2-propanol/water/acetic acid/Trimethylamine (85:14:1:0.08 v/v/v/v) was applied for 24 min. Neutral lipids were analyzed in isocratic regime in the direct phase column applying hexane/2-propanol/acetic acid (90:15:1 v/v/v) as the solvent. The flow rate was 1 ml/min. The data were processed using Empower software, and the ELSD was regularly calibrated using commercial high-purity standards for each lipid.

Triacylglyceride (TAGs) were purified from the neutral lipid fraction by (TLC) for fatty acid composition determination by gas chromatography coupled to a flame ionization detector (GC/FID). Neutral lipid fractions were applied to TLC Silica gel 60 20 × 20 cm (Merck Milipore) previously activated at 80°C for 60. Each lane of the TLC plate was loaded either with 200 μg of sample or with 50 μg of specific lipid standard. The TLC solvent system used was hexane/diethyl ether/acetic acid 70:30:1 (v/v). Lipid bands on the TLC plates were detected by spraying with a solution of primuline (0.05%, w/v) in acetone/water 80:20 (v/v) and scrapped off from the plate. Lipids in the silica gel were methylated with 2 ml of hydrogen chloride (HCl)-methanol (1.25 M HCl) (Sigma) at 80°C for 1 h. Fatty acid methyl esters (FAMEs) were then extracted with 3 ml of hexane (Sigma). Then, the FAMEs were concentrated to dryness by removing the solvent under a stream of nitrogen and then re-suspended in a volume of 150 μl of heptane (Sigma) and transferred to a vial with an insert for GC/FID analysis. The samples were analyzed in a gas chromatograph (Perkin Elmer Clarus 500) equipped with a 0.2 μm × 60 m × 0.25 mm fused silica capillary column, hydrogen at 45 ml min^–1^ was employed as carrier gas and coupled to a flame ionization detector. The oven was programmed for an initial temperature of 140°C (hold for 2 min), followed by an increase of 10°C min^–1^ to 210°C (hold for 7 min). Also set were the following parameters: 270°C inlet and 280°C detector temperatures. To assign the peaks in the spectral data, a standard mixture with known composition of FAMEs (Sigma) was previously run in the same conditions, and the assignment was done based on the retention time and the area of the peaks.

### Microscopy Analyses

Synchronized animals were grown until the appropriate stage and then mounted on 2% agarose pads with 10 mM Levamisole to be imaged (10–30 worms in each assay with at least 2 independent experiments). The UPR^ER^ (*Phsp-4:gfp*) and vitellogenesis (*Pvit-2:vit-2:gfp* and *Pvit-6:vit-6:gfp*) were assessed using an AxioCam MRm camera on a Zeiss ApoTome Microscope. Lipid droplet coverage (*Pvha-6:dgat-2:gfp*) was analyzed using a Confocal Microscope Leica SP2-AOBS. The UPR^ER^ (*Phsp-4:gfp*) on *phb-1(tm2751)* mutants, the DNJ-27 reporter and ATGL-1 expression (*Patgl-1:atgl-1:gfp)* was assessed using an ORCA-Flash4.0 LT Hamamatsu digital camera on a Leica M205 Stereoscope equipped with a Plan Apo 5.0x/0.50 LWD objective. Image analyses was performed using the ImageJ software. For the UPR^ER^, vitellogenesis and ATGL-1 expression analyses, worms were manually segmented and the mean GFP intensity per worm was calculated. For DNJ-27 reporter the head of the animals was exclude from the analysis. Quantification of the LD intestinal coverage was done in the anterior part of the intestine (int1 and int2 intestinal cells). LDs were segmented in ImageJ and classified as smaller (less than 1 μm^2^) and larger (equal or bigger that 1 μm^2^). Data was analyzed using the GraphPad Prism software.

### Electron Microscopy

Transmission electron microscopy (TEM) was carried out as described (Hall et al., 2012) with small modifications. Adult worms at day 1 were immersed in 0.8% glutaraldehyde + 0.8% osmium tetroxide in 0.1 M sodium cacodylate buffer, pH 7.4. Under a dissecting scope, animals were cut with a scalp at the posterior end of the intestine (removing the tail) and kept for 1 h on ice under dark conditions. Worms were washed three times in 0.1 M sodium cacodylate buffer, fixed overnight (2% osmium tetroxide in 0.1 M sodium cacodylate buffer), washed three times more in the same buffer (all previous steps were performed on ice) and finally embedded in small cubes of 1% agarose. Next, worms were dehydrated at room temperature by incubating them in 50%, 70%, and 90% ethanol (10 min each), followed by three washes (100% ethanol and 10 min each). Worms were then incubated for 15 min in an ethanol/propylene oxide solution (50:50 v/v), followed by two incubations (15 min) in 100% propylene oxide. After, worms were infiltrated on a rotator or with an embedding machine, first for 2 h in a 3:1 ratio of propylene oxide to resin and then for 2 h in a 1:3 ratio of propylene oxide to resin, incubated overnight in 100% resin and followed by 4 h incubation in fresh 100% resin. Finally, worms were arranged in a flat embedding mold and cured at 60°C for 2 days. For TEM imaging, worms were cut at the anterior part, at approximately 350 μm from the mouth (at least two different animals per condition). Image analysis was performed with the ImageJ software, where the worm intestine and intestinal LDs were manually segmented, and the areas measured. Data was analyzed using the GraphPad Prism software.

### Lifespan Analysis

Lifespan assays were initiated by allowing gravid adults to lay eggs on NGM plates seeded with the previously prepared bacterial stocks. Worms were transferred every day throughout their reproductive period and every 2–4 days thereafter. Animals were scored as dead when they stopped responding to touch, while ruptured animals or those that suffered internal hatching, extruded gonad or desiccation were censored in the data analysis. Data was analyzed using the GraphPad Prism software.

### Data Availability

Tabulated lipidomics data is available at: https://data.mendeley.com/datasets/dvvp4f25y6/1 (Lourenço et al., 2020).

## Results

### The Mitochondrial Prohibitin Complex Has a Stronger Effect on the Lipidome of Wild Type Worms Than of *daf-2(e1370)* Mutants

The impact of the PHB on the *C. elegans* lipidome was assessed, in wild type animals and *daf-2* mutants, by depleting *phb-1* using RNA interference (RNAi). PHB-1 and PHB-2 proteins are interdependent for protein complex formation, therefore, depletion of *phb-1* results in lack of the complete PHB complex (Artal-Sanz et al., 2003; Lourenco et al., 2015; Hernando-Rodriguez et al., 2018). Additionally, PHB-1 and PHB-2 are equally expressed and decrease to the same extent upon *phb-1(RNAi)* both in wild type and *daf-2* mutant animals (Gatsi et al., 2014). Principal component analysis (PCA) of the LC-MS lipid profiles evidenced a clear clustering pattern (Supplementary Figures 1A,B). Namely, a separation based on genetic backgrounds (wild type versus *daf-2* mutant) and on the effect of depleting PHB in either of the genetic backgrounds (Supplementary Figure 1A). Hierarchical clustering analysis indicated that *phb-1(RNAi)* had a more pronounced effect in the LC-MS profiles of wild type worms than of *daf-2* mutants (Supplementary Figure 1B). Yet, the LC-MS profiles of *daf-2* and *daf-2;phb-1(RNAi)* were still clearly distinguishable (Supplementary Figures 1A,B). In the following sections, we describe in more detail the more relevant lipid species found to have their content altered due to PHB deficiency and/or *daf-2* mutation (see tabulated lipidomics dataset at https://data.mendeley.com/datasets/dvvp4f25y6/1).

### PHB Deficiency and *daf-2* Mutation Differentially Affect the Composition of Triacylglycerides

**FIGURE 1:**
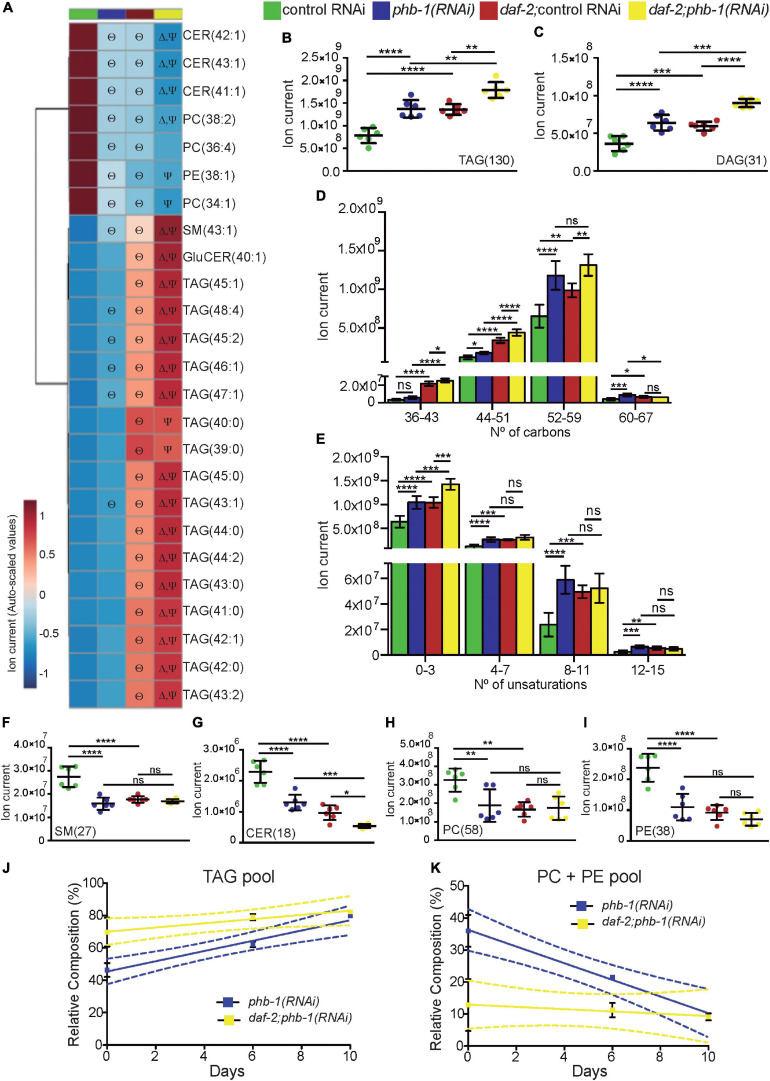
Glycerolipid and glycerophospholipid pools are affected by PHB depletion in a genetic background dependent-manner. **(A)** Heat map displaying the top 25 lipid species, ranked based on the P-values coming from One-way ANOVA with *post hoc* Tukey multiple comparison test, responding to PHB deficiency and/or to *daf-2(e1370)* mutation. Statistical significance compared to control RNAi (Θ), *daf-2(e1370)* mutation (Δ) or *phb-1(RNAi)* (Ψ). **(B,C)** Quantification of the pools of TAG **(B)** and DAG **(C)** based on the LC-MS data acquired in the positive mode. The quantification corresponds to the sum of the ion current of all individual lipid species (between brackets are the number of lipid species) shown to be relevant for cluster separation in all pair-wise comparisons. **(D)** Quantification of the TAG pools accordingly to the total carbon number, given by the sum of the three radyl side-chains carbon number. **(E)** Quantification of the TAG pools accordingly to the number of total unsaturations, given by the sum of the three radyl side-chains unsaturation number. Mean values from six independent biological replicas; error bars denote SD. One-way ANOVA with *post hoc* Tukey multiple comparison test, *P*-value < 0.05 (*) < 0.01 (**) < 0.001 (***) < 0.0001 (****). **(F–I)** Quantification of the pools of **(F)** SM, **(G)** CER, PC **(H)** and PE **(I)** based on the LC-MS data acquired in the positive mode (see **B,C**). Mean values from six independent biological replicas; error bars denote SD. One-way ANOVA with *post hoc* Tukey test. *P*-value < 0.05 (*) < 0.01 (**) < 0.001 (***) < 0.0001 (****). **(J,K)** HPLC analysis of TAG **(J)** and PC + PE **(K)** during ageing (days 0, 6, and 10) in wild type and *daf-2* mutants upon PHB depletion. The lipid relative composition expresses the percentage of a given lipid species within the total lipid fraction. Mean values from at least two independent biological replicates of each condition [except for *phb-1(RNAi)* in wild type worms at day 10, where data from only one replicate was collected]; error bars denote SD. Dashed lines depict the 95% interval of confidence of the linear regression. TAG, Triacylglycerol; DAG, Diacylglycerol; SM, Sphingomyelin; CER, Ceramide; PC, Phosphatidylcholine; PE, Phosphatidylethanolamine.

Similar to *daf-2* mutants (Ogg et al., 1997; Perez and Van Gilst, 2008; Prasain et al., 2015), PHB depletion increased the content of the large majority of glycerolipids, further increasing diacylglyceride (DAG) and TAG pools in *daf-2* mutants (Figures 1A–C). Interestingly, all the TAG species within the Top25 relevant lipids (ranked by P values) had a low/medium total carbon number (38–48 carbons) and a low total number of unsaturations. Moreover, most of the statistically significant changes occurred in the context of *daf-2* mutants (Figure 1A). Looking at the TAG composition more in detail, PHB depletion in otherwise wild type animals led to a slight increase in TAGs with a low total carbon number but a strong increase, similar to the effect of *daf-2* mutants, in TAGs with a high total carbon number (Figure 1D). Indeed, in wild type worms, PHB depletion mostly increased the amount of TAG species with a high total carbon number, while in *daf-2* mutants the distribution mode was clearly shifted toward to TAGs with lower total carbon number (Supplementary Figure 1C). Additionally, while PHB depletion in otherwise wild type animals increase TAGs irrespective of the total number of unsaturations, in *daf-2* mutants the increase was restricted to TAGs with a low of unsaturations (Figure 1E and Supplementary Figure 1D). In line with these findings, analysis of fatty acid composition from whole worm TAG, after thin layer chromatography (TLC), showed that PHB-depleted animals had less shorter monounsaturated fatty acids compared to PHB-depleted *daf-2* mutants, where longer saturated fatty acids where increased (Supplementary Figure 1E and Supplementary Table 1). Taken together, we conclude that while both PHB deficiency and *daf-2* mutants increase the TAG pool, they trigger a differential effect in the TAG pool composition, suggesting differential mechanisms in the balancing and mobilization of the TAG pool.

### PHB Depletion Alters the Pools of Membrane Lipids in a Genetic-Background-Dependent Manner

Membrane lipids are important in a diversity of cellular functions and have been shown to alter their amounts during ageing (Hou and Taubert, 2012; Egawa et al., 2016; Jove et al., 2020). Our LC-MS analysis revealed that, at the YA stage, many glycerophospholipids and sphingolipids species changed their content upon PHB depletion and/or in response to *daf-2* mutation, being some of them among the Top25 relevant lipid species (Figure 1A). Different sphingolipids like sphingomyelin (SM) and ceramide (CER) were perturbed as a result of PHB depletion and/or *daf-2* mutation (Figures 1F,G). Importantly, phosphatidylcholine (PC) and phosphatidylethanolamine (PE) pools, the two major glycerophospholipids pools and important membrane lipids (Satouchi et al., 1993; Vrablik et al., 2015), decreased in response to PHB depletion (Figures 1H,I). As previously reported (Prasain et al., 2015), *daf-2* mutation decreased the PC and PE pools, while PHB depletion in *daf-2* mutants had a negligible effect. The observed differential effect of PHB deficiency in membrane lipids in wild type animals and in *daf-2* mutants could impact differentially the functionality of organelles and the physiology of the animals during ageing.

### Glycerolipid and Glycerophospholipid Pools Evolve Sharply During Ageing Specifically in PHB-depleted Worms

Collectively, our lipidomics data suggested the existence of a balance between glycerolipids (TAG) and glycerophospholipids (PE + PC) pools upon PHB depletion in otherwise wild type worms. A complementary HPLC analysis evidenced that in the absence of PHB, TAG and glycerophospholipid (PE + PC) pools change in a genetic-background-dependent manner during chronological ageing (Figures 1J,K). Assuming a linear relationship with time (days), the respective linear coefficients for TAG and PE + PC pools were 3.316 (*R*^2^ = 0.950) and −2.603 (*R*^2^ = 0.949) for PHB-depleted worms and 1.297 (*R*^2^ = 0.560) and −0.356 (*R*^2^ = 0.103) for PHB-depleted *daf-2* mutants. The TAG pool in PHB-depleted animals increased during ageing while in PHB-depleted *daf-2* mutants it was much less affected (F test *P*-value 0.036). Oppositely, the PE + PC pool in PHB-depleted animals decreased during ageing while in PHB-depleted *daf-2* mutants it was much less affected (F test *P*-value 0.010). Altogether, our data indicates that the lipidome of PHB-depleted worms is more prone to changes during ageing than that of PHB-depleted *daf-2* mutants.

### PHB-depletion Dysregulates Yolk Homeostasis in a Genetic Background Dependent-Manner

**FIGURE 2:**
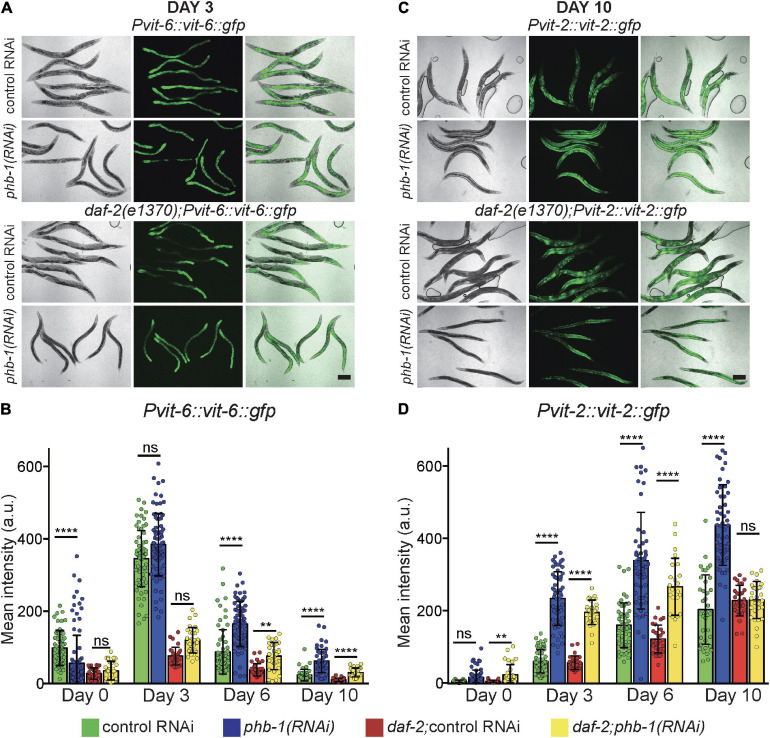
PHB deficiency dysregulates yolk homeostasis in a genetic background dependent-manner. **(A)** Representative images of VIT-6:GFP at day 3 of adulthood and **(B)** respective quantification of the mean GFP intensity per worm during ageing; error bars denote SD. **(C)** Representative images of VIT-2:GFP at day 10 of adulthood and **(D)** respective quantification of the mean GFP intensity per worm during ageing; error bars denote SD. At least two independent biological replicates of each condition were performed. One-way ANOVA-Dunn’s multiple comparisons test. *P*-value < 0.01 (**) < 0.0001 (****). Scale Bars: 200 μm.

Driven by the results of our untargeted lipidomic analysis, we focus on the effect of PHB depletion on organelles where most TAG accumulate, LD and yolk/lipoprotein particles. Vitellogenesis has a major impact on the worm lipid homeostasis, particularly during the reproductive period where a substantial fraction of the somatic lipid content is channeled for yolk production. In worms three vitellogenins generate distinct yolk lipoprotein complexes. *vit-6* gene product, cleaved after secretion into the pseudocoelom, is the sole source of YP115 and YP88 (Sharrock et al., 1990). Due to the characteristics of the reporter, VIT-6:GFP is visible almost exclusively in the intestine where it is produced (Supplementary Figure 2). VIT-6, peaked during the reproductive period and decreased during ageing (Figures 2A,B). Prohibitin complex deficiency did not alter the overall dynamics, however, it increased VIT-6 levels in 6 and 10 days-old adults in both genetic backgrounds, while *daf-2* mutants reduced VIT-6 levels (Figure 2A) (*P*-value < 0.0001 and <0.01, respectively). Next, we focused on *vit-2*, one of the sources of the YP170 pool (Sharrock et al., 1990), which has been specifically associated with intestinal atrophy and aging (Sornda et al., 2019). VIT-2:GFP was visible in different parts of the worm body, including the intestine, where it is produced, the gonad, where it is translocated to oocytes, and the body cavity where it accumulates during ageing (Seah et al., 2016) (Figure 2C and Supplementary Figure 2). PHB-depletion increased VIT-2 accumulation during ageing, from the YA stage to day 10 of adulthood (Figures 2C,D). Interestingly, in *daf-2* mutants, PHB-depletion increased VIT-2 levels coincident with the reproductive period but not in old (day 10) animals (Figures 2C,D). Aged PHB depleted otherwise wild type animals accumulated larger amounts of displaced yolk through the worm body with a concomitant accumulation of different lipids.

### PHB and DAF-2 Differently Affect Lipid Droplet Biology

**FIGURE 3:**
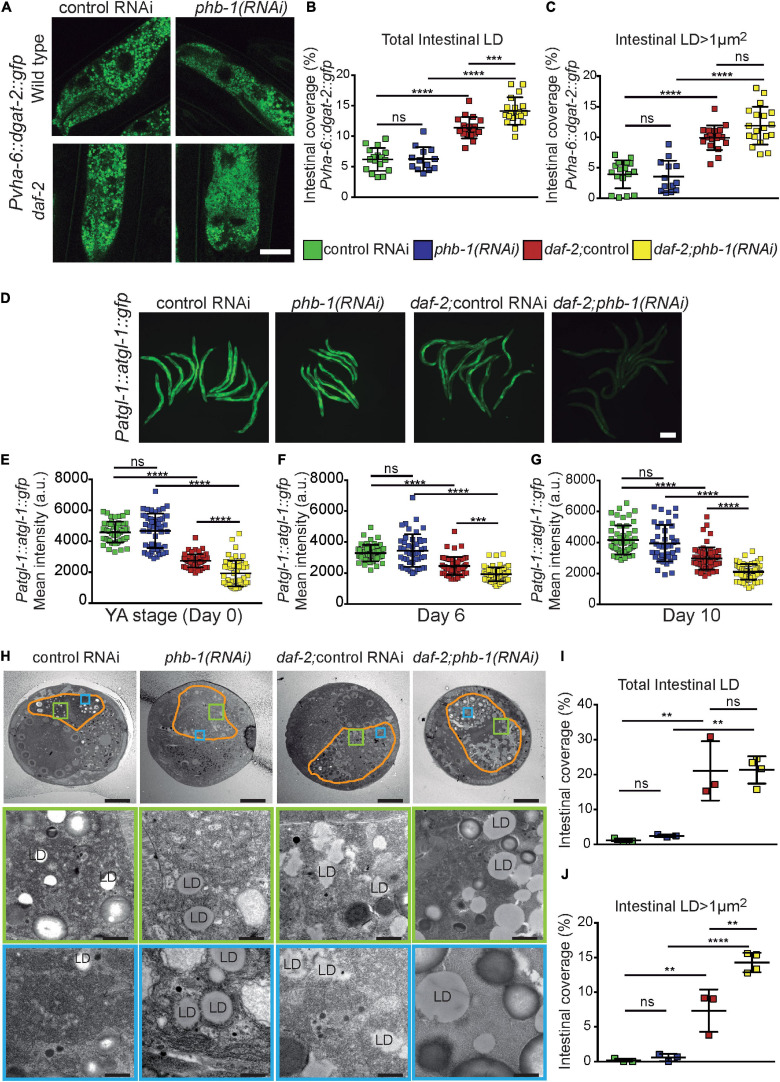
PHB deficiency and/or *daf-2(e1370)* mutation differentially affect lipid droplets. **(A)** Representative images of DGAT-2:GFP-labeled lipid droplets (LD) at the YA stage (Scale Bar: 20 μm). **(B,C)** Quantification of LD coverage (%) given by the ratio between the sum of the area of intestinal LDs versus the worm intestinal area times 100. All LDs **(B)** and LDs larger than 1 μm^2^ **(C)**. Mean values from at least two independent biological replicates; error bars denote SD. ANOVA with *post hoc* Tukey test. *P*- values < 0.001 (***) < 0.0001 (****). **(D)** Representative images of ATGL-1:GFP at the YA stage. Scale Bar: 200 μm. **(E–G)** Quantifications of the mean intensity GFP signal per worm at days 0 (YA stage) **(E)**, 6 **(F)**, and 10 **(G)** of adulthood. Mean of at least two independent biological replicates; error bars denote SD. One-way ANOVA with *post hoc* Tukey multiple comparison test or, when appropriate, one-way Kruskal-Wallis test with *post hoc* Dunn’s Multiple comparison test, *P*-value < 0.05 (*) < 0.01 (**) < 0.001 (***) < 0.0001 (****). **(H)** Representative electron micrographs (EM) of worm cross-sections (top panels) and intestinal regions (middle and bottom panels) of wild type worms, PHB-depleted animals, *daf-2* mutants and PHB-depleted *daf-2* mutants at day 1 of adulthood. The intestine is delineated in orange. Bar sizes: 10 μm (top panels), 1 μm (middle panels) and 500 nm (bottom panels). **(I,J)** Quantification of intestinal LD coverage of all LDs **(I)** and LDs larger than 1μm^2^ **(J)** in each intestinal EM cross-section analyzed. At least three EM cross sections of the complete intestine were analyzed per genotype. One-way ANOVA with *post hoc* Tukey multiple comparison test, *P*-value < 0.01 (**) < 0.0001 (****).

Lipids droplets (LDs) are ubiquitous fat storage organelles where neutral fat such as TAG are accumulated and mobilized according to the organism needs for membrane synthesis and energy. Using worms expressing GFP-tagged diacyl glycerol acyltransferase-2 (DGAT-2), a LD-associated protein (Xu et al., 2012), we found that *daf-2* mutants had higher LD intestinal coverage than wild type animals at the YA stage, while PHB-depletion had no effect (Figures 3A–C and Supplementary Figures 3A,B). The effect of *daf-2* mutation was associated with an increase on the intestinal coverage of larger LDs (Figure 3C and Supplementary Figures 3A,B). Additionally, PHB-depletion in *daf-2* mutants increased DAGT-2-labeled LD coverage (Figures 3A–C and Supplementary Figures 3A,B), being this also associated with an increase in the intestinal coverage of larger LDs (unpaired *t*-test P-value 0.027). We then explored the impact of PHB on ATGL-1, an important lipase for LD TAG mobilization (Zhang et al., 2010; Kong et al., 2020). ATGL-1 levels were always higher in wild type worms than in *daf-2* mutants during ageing. Interestingly, while PHB depletion did not affect ATGL-1 levels in wild type animals, it consistently lowered ATGL-1 levels in *daf-2* mutants (Figures 3D–G). Finally, we measured the LD intestinal coverage by TEM analyses at day 1 of adulthood. Consistently, *daf-2* mutants increased LD intestinal coverage compared to wild type animals, irrespective of considering total, smaller or larger LDs, (Figures 3H–J and Supplementary Figures 3C,D). The effect of PHB-depletion in otherwise wild type animals was weaker than the effect of *daf-2* mutation (Figures 3H–J and Supplementary Figures 3C,D). Still, considering total LDs, PHB-depletion significantly increased LD intestinal coverage (unpaired *t*-test *P*-value 0.019). Interestingly, in a *daf-2* mutant background, PHB further increased the intestinal coverage of larger LDs (Figures 3H,J and Supplementary Figure 3E). All these data show that, while PHB depletion in *daf-2* mutants synergistically increased the LD intestinal coverage of larger LDs, on their own PHB and DAF-2 differently affect LD homeostasis.

### PHB Deficiency Affects the UPR^ER^ Differently in Conditions Leading to Opposing Longevity Phenotypes

**FIGURE 4:**
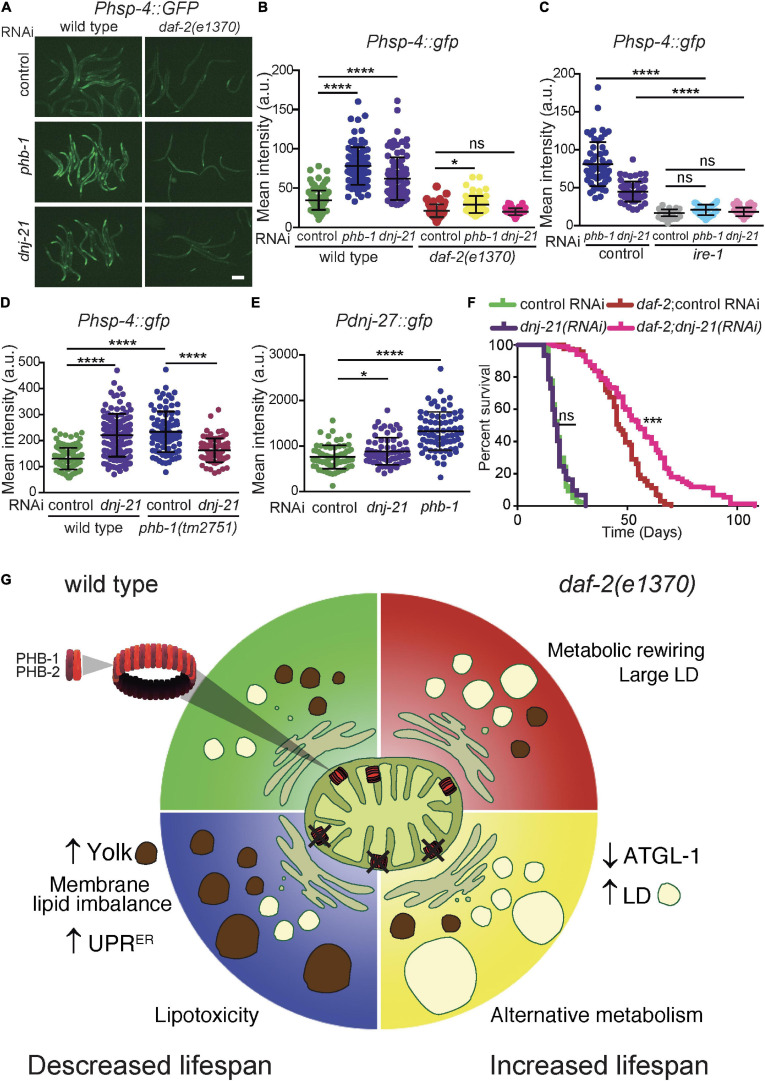
PHB and DNJ-21 deficiency have a similar effect on the UPR^ER^ stress response and on lifespan. **(A,B)** Representative images **(A)** and quantification **(B)** of the UPR^ER^ stress reporter P*hsp-4:GFP* at the YA stage in wild type and *daf-2* genetic backgrounds upon *phb-1* and *dnj-21* RNAi. Scale Bar: 200 μm. **(C)** Effect of *ire-1(RNAi)* on the induction of the UPR^ER^ stress response in PHB-1 and DNJ-21-depleted animals. Control RNAi is shown in panel B. ANOVA-Dunn’s multiple comparisons test, *P*-value < 0.05 (*), < 0.0001 (****), ns non-significant. Mean values from at least two independent biological replicas; error bars denote SD. **(D)** Quantification of the P*hsp-4:GFP* stress reporter in *phb-1(tm2751)* deletion mutants upon *dnj-21(RNAi)* at the early-L4 larval stage. Mean of three independent replicas. ANOVA-Dunn‘s multiple comparisons test, *P*-value < 0.0001 (****). **(E)** Quantification of the induction of the P*dnj-27*:GFP by *phb-1(RNAi)* or *dnj-21(RNAi)* at the YA stage. The head region was excluded from the analysis (see Supplementary Figure 4C). Mean values from three independent replicas are shown, error bars are SD. Mann-Whitney test. *P*-value < 0.05 (*), < 0.0001 (****). **(F)** Lifespan analysis on the effect of *dnj-21(RNAi)* in wild type and *daf-2* genetic backgrounds. Combined lifespan data from at least two independent experiments is presented (20°C). Lifespan curves are represented as the percentage of animals remaining alive against animal age (days). Log-rank (Mantel-Cox) test was used to derive statistical significance (*P*-value < 0.001 (***), ns non-significant). See Supplementary Table 2 for the data of independent assays **(G)** Model on how changes in the worm lipidome upon PHB depletion might lead to opposing effects on longevity.

A critical organelle in lipid homeostasis is the ER. Specifically, the ER is important in the balance between glycerophospholipid and glycerolipids (Jacquemyn et al., 2017; Watts and Ristow, 2017) and for the formation of LDs (Jacquemyn et al., 2017; Watts and Ristow, 2017; Cao et al., 2019; Olzmann and Carvalho, 2019) and yolk particles (Seah et al., 2016). Therefore, we assessed the impact of PHB on the ER using the UPR^ER^ stress reporter *Phsp-4:GFP*. At the YA stage, PHB depletion in otherwise wild-type animals induced the UPR^ER^ while *daf-2* mutants were protected (Figures 4A,B). In mammals, PHB interacts with DNAJC19, a co-chaperone for mitochondrial protein import, to regulate cardiolipin remodeling in the mitochondrial inner membrane (Richter-Dennerlein et al., 2014). Similar to PHB depletion, lack of the *C. elegans* DNAJC19 ortholog, DNJ-21, induced the UPR^ER^ stress reporter, being *daf-2* mutants protected (Figures 4A,B). To check if UPR^ER^ induction upon mitochondrial depletion of PHB-1 and DNJ-21 was dependent on *ire-1*, a key UPR activator (Gardner et al., 2013), we simultaneously depleted *ire-1* together with either *phb-1* or *dnj-21* using double RNAi. The efficiency of the double RNAi was evident as developmental delay and reduced body size was observed upon depletion of both mitochondrial genes, as with full RNAi (data not shown). We observed that IRE-1 is required to mount the UPR^ER^ in PHB-1 and DNJ-21 deficient animals (Figure 4C, control RNAi is the same as in panel B). To assess the interaction of PHB and DNJ-21 in the regulation of the UPR^ER^ we introduced the *Phsp-4:GFP* reporter in a *phb-1* deletion mutant. Homozygous *phb-1* mutants develop into sterile adults due to maternal contribution (Artal-Sanz and Tavernarakis, 2009b; Hernando-Rodriguez et al., 2018). However, depletion of *dnj-21* arrested development of *phb-1* mutants at the late L3/L4 larval stage (Supplementary S4 A and B) and partially reduced the UPR^ER^, probably due to the synthetic interaction (Figure 4D and Supplementary Figures 4A,B). To further confirm induction of the UPR^ER^, we tested the expression of a DNJ-27 reporter at the YA stage (Figure 4E), an ER luminal protein induced upon ER stress via IRE-1 (Munoz-Lobato et al., 2014). Indeed, while depletion of DNJ-21 mildly increased the expression of the reporter, lack of PHB resulted in a clear induction of DNJ-27 (Figure 4E and Supplementary Figure 4C). We then asked if DNJ-21 depletion could mimic the opposing longevity phenotype of PHB depletion. Interestingly, we found that *dnj-21(RNAi)* extended the lifespan of *daf-2* mutants but not wild type animals (Figure 4F and Supplementary Table 2). These results suggest a connection between ER functionality and the opposing effect of PHB depletion in the longevity of wild type and insulin signaling mutant animals.

## Discussion

We previously showed that the mitochondrial PHB affects fatty acid metabolism in *C. elegans* in a genetic-background-specific manner (Lourenco et al., 2015). Interestingly, we found that *daf-2* mutation and PHB deficiency alters the TAG composition in a clear distinct manner. Similarly, membrane phospholipids (PLs) are reduced upon PHB depletion but not in *daf-2* mutants, where PLs are generally reduced. Moreover, our results suggest the existence of a negative association between TAG and PL pools in PHB-depleted animals. Indeed, during chronological ageing, TAG increase and PL decrease in a much more pronounced manner in PHB-depleted otherwise wild type worms than in PHB-depleted *daf-2* mutants. This suggests that lipid metabolic pathways, anabolic and/or catabolic, tuned by PHB depletion are distinct depending on the genetic background and different from the ones triggered by the *daf-2* mutation.

Intestinal cells, as well as oocytes and embryos within the germline, are key sites where TAG accumulate in *C. elegans* (Hall et al., 1999). In fact, a substantial fraction of the somatic lipid content of gravid animals is yolk-related (Kimble and Sharrock, 1983; Grant and Hirsh, 1999). Yolk is produced at the expenses of the gut by a process of general autophagy, which is detrimental in the mid/long term (Ezcurra et al., 2018). Indeed, YP170/VIT-2 has been proposed as a major driver of worm senescence (Sornda et al., 2019). Additionally, although still a matter of controversy, perturbation of vitellogenesis leads to yolk steatosis and to lipotoxicity with a negative impact on ageing (Ackerman and Gems, 2012; Steinbaugh et al., 2015; Seah et al., 2016; Palikaras et al., 2017; Perez and Lehner, 2019). Wild type worms accumulate yolk with a concomitant increase of the TAG pool during ageing (Ezcurra et al., 2018). Curiously, while PHB depletion leads to sterility (Artal-Sanz et al., 2003; Artal-Sanz and Tavernarakis, 2009b), it does not switch off vitellogenesis. Instead, yolk accumulates massively in aged worms, as in a different sterility condition (Steinbaugh et al., 2015). Strikingly, this increase, passed the reproductive period, is fully suppressed by *daf-2* mutation, presumably due to a downregulation of vitellogenesis (DePina et al., 2011) and/or to lipophagy as in germline-less worms (Lapierre et al., 2011). In the mid-life *daf-2* mutants display hypometabolic features. Particularly, *daf-2* mutants repress major metabolic pathways namely associated to lipid metabolism including vitellogenesis (Halaschek-Wiener et al., 2005). Interestingly, the balance of yolk-related lipids, which would otherwise be stored and/or remodeled in the intestine, has been described to play a role in intestinal lipid homeostasis and life span extension (Seah et al., 2016). PHB-depleted *daf-2* mutants have larger intestinal LDs and lower expression of ATGL-1 than *daf-2* mutants. ATGL-1, the worm homolog of the mammalian rate-limiting lipolytic enzyme ATGL, is required for *daf-2* mutants’ longevity (Zaarur et al., 2019). Hence, it is tempting to speculate that in PHB-depleted *daf-2* mutants the excess of TAGs is mainly accommodated in larger intestinal LDs, which are mobilized during ageing through tighter regulation of ATGL-1. In parallel, the dysregulation in lipid and yolk pools occurring earlier in PHB-depleted worms is what determines a faster ageing pace (Figure 4G).

Interestingly, recent research reveals the importance of mitochondrial contacts with the rough ER for lipoprotein secretion and systemic lipid homeostasis (Anastasia et al., 2021). Similarly, it has long been recognized the importance of the interaction between mitochondria and the smooth ER, through mitochondria-associated ER membranes (MAM), for synthesis and maturation of key membrane lipids including phospholipids and sphingolipids (Vance, 2014). Membrane lipid composition is a key determinant of structural and functional integrity of eukaryotic membrane-bound organelles (van Meer et al., 2008; Antonny et al., 2015). Indeed, dysregulation of membrane lipid composition leads to lipotoxicity (Hou et al., 2014; Antonny et al., 2015). Although organelle-specific lipidomics data will be key to assess the impact of PHB on the PL and sphingolipids membrane composition, here we show a differential effect of PHB depletion in whole worm PL and sphingolipids levels in wild type versus *daf-2* mutants. Sphingolipids are essential components of biological membranes, that together with ceramides regulate intracellular trafficking, signaling and stress responses. In worms, sphingolipid biosynthesis has been linked to the UPR^mt^, as phosphorylated sphingosine is required in mitochondria to activate the UPR^mt^ (Kim and Sieburth, 2018). Inhibition of sphingolipids biosynthesis reduces induction of the UPR^mt^ upon mitochondrial stress due to lack of ceramide (Liu et al., 2014). Thus, it is possible that reduced ceramide levels in *daf-2* mutants contribute to the attenuated UPR^mt^ upon PHB depletion (Gatsi et al., 2014). Moreover, reduced sphingolipid and ceramide synthesis extend lifespan of worms and flies (Johnson and Stolzing, 2019). These phenotypes resemble the effect of *daf-2* mutation in PHB depleted animals; reduced UPR^mt^ induction and increased lifespan (Artal-Sanz and Tavernarakis, 2009b; Gatsi et al., 2014). Interestingly, we have recently shown that PHB is required for the lifespan extension conferred by reduced sphingolipid synthesis in *C. elegans* (de la Cruz-Ruiz et al., 2021). It would be interesting to further evaluate the role of sphingolipid metabolism in the lifespan extension conferred by PHB depletion to *daf-2* mutants.

For the first time, we show that PHB deficiency induces the UPR^ER^, suggesting a dysregulation in the interaction between mitochondria and ER, in a genetic background-dependent manner. *daf-2* mutant animals are protected against ER stress, which has been linked to its longevity phenotype (Henis-Korenblit et al., 2010; Zhou et al., 2011). Prohibitin complex deficiency has been previously claimed not to affect the UPR^ER^ (Bennett et al., 2014). However, although PHB-depletion did not further induce the UPR^ER^ in the presence of tunicamycin, an ER stressor, in its absence *phb-2(RNAi)* induced the UPR^ER^ (Bennett et al., 2014). Moreover, PHB overexpression has been recently shown to block ER stress in a mice model of Parkinson’s disease (Wang et al., 2021). Importantly, PHB genetically interacts with genes involved in mitochondria-ER contact sites (Kornmann et al., 2009), which are important for transferring PLs (Senft and Ronai, 2015; Phillips and Voeltz, 2016). Indeed, lipid perturbation has been shown to activate the UPR^ER^ [reviewed in Xu and Taubert (2021)]. In mammalian cells, PHB was found to interact with DNAJC19, the mitochondrial mtHsp70 co-chaperone, in cardiolipin remodeling (Richter-Dennerlein et al., 2014). Depletion of DNJ-21, the worm homolog of DNAJC19, and of PHB induces the UPR^mt^ to the same magnitude, as assessed by *Phsp-6:gfp* (Bennett et al., 2014), where HSP-6 is the worm ortholog of mtHsp70. Interestingly, DNJ-21 depletion also induces the UPR^ER^ (Bennett et al., 2014). Indeed, DNJ-21 depletion triggers the UPR^ER^ in a genetic background dependent-manner as PHB depletion does. Importantly, while DNJ-21 depletion either does not affect or shortens (Bennett et al., 2014) the lifespan of otherwise wild type animals, feeding *daf-2* mutant animals with *dnj-21(RNAi)* markedly extends lifespan. Altogether it reinforces the idea that PHB, which has been proposed to be a chaperone (Nijtmans et al., 2002 #24;Nijtmans et al., 2000 #20) and DNJ-21 functionally interact, at least partially, to modulate mitochondria and ER function. Importantly, it provides for the first time, data supporting a link between the ER and PHB in determining the differential ageing phenotype.

## Conclusion

This work brought extremely valuable new insights in the remodeling occurring in the worm lipidome upon PHB depletion. Importantly, PHB depletion resonates in other mitochondria close interacting organelles. Strikingly, PHB depletion leads to a differential dysregulation of the ER in wild type animals and long-lived insulin mutants. Our work opens the door to further explore the importance of the interaction between ER and mitochondria in the opposing effect of PHB depletion in longevity.

## Data Availability Statement

The lipidomics datasets presented in this study can be found in online repositories. The names of the repository/repositories and accession number(s) can be found below: https://data.mendeley.com/datasets/dvvp4f25y6/1.

## Author Contributions

MA-S conceived the idea. AL and MA-S designed the experiments and wrote the manuscript. AL, DC, MD, PW, MV-C, JS, BH-R, and MR-P carried out the experiments. AL, MR-P, and MA-S analyzed data and interpreted results. All authors contributed to the article and approved the submitted version.

## Conflict of Interest

The authors declare that the research was conducted in the absence of any commercial or financial relationships that could be construed as a potential conflict of interest.

## References

[B1] Ackerman D. Gems D. (2012). The mystery of *C. elegans* aging: an emerging role for fat. Distant parallels between *C. elegans* aging and metabolic syndrome? *Bioessays* 34 466–471. 10.1002/bies.201100189 22371137

[B2] Anastasia I. Ilacqua N. Raimondi A. Lemieux P. Ghandehari-Alavijeh R. Faure G. (2021). Mitochondria-rough-ER contacts in the liver regulate systemic lipid homeostasis. *Cell Rep.* 34:108873. 10.1016/j.celrep.2021.108873 33730569

[B3] Antonny B. Vanni S. Shindou H. Ferreira T. (2015). From zero to six double bonds: phospholipid unsaturation and organelle function. *Trends Cell Biol.* 25 427–436. 10.1016/j.tcb.2015.03.004 25906908

[B4] Artal-Sanz M. Tavernarakis N. (2009a). Prohibitin and mitochondrial biology. *Trends Endocrinol. Metab.* 20 394–401. 10.1016/j.tem.2009.04.004 19733482

[B5] Artal-Sanz M. Tavernarakis N. (2009b). Prohibitin couples diapause signalling to mitochondrial metabolism during ageing in *C. elegans*. *Nature* 461 793–797. 10.1038/nature08466 19812672

[B6] Artal-Sanz M. Tavernarakis N. (2010). Opposing function of mitochondrial prohibitin in aging. *Aging (Albany NY.)* 2 1004–1011. 10.18632/aging.100246 21164222 PMC3034168

[B7] Artal-Sanz M. Tsang W. Y. Willems E. M. Grivell L. A. Lemire B. D. van der Spek H. (2003). The mitochondrial prohibitin complex is essential for embryonic viability and germline function in *Caenorhabditis elegans*. *J. Biol. Chem.* 278 32091–32099. 10.1074/jbc.M304877200 12794069

[B8] Back J. W. Sanz M. A. De Jong L. De Koning L. J. Nijtmans L. G. De Koster C. G. (2002). A structure for the yeast prohibitin complex: Structure prediction and evidence from chemical crosslinking and mass spectrometry. *Protein Sci.* 11 2471–2478. 10.1110/ps.0212602 12237468 PMC2373692

[B9] Bennett C. F. Vander Wende H. Simko M. Klum S. Barfield S. Choi H. (2014). Activation of the mitochondrial unfolded protein response does not predict longevity in *Caenorhabditis elegans*. *Nat. Commun.* 5:3483. 10.1038/ncomms4483 24662282 PMC3984390

[B10] Cao Z. Hao Y. Fung C. W. Lee Y. Y. Wang P. Li X. (2019). Dietary fatty acids promote lipid droplet diversity through seipin enrichment in an ER subdomain. *Nat. Commun.* 10:2902. 10.1038/s41467-019-10835-4 31263173 PMC6602954

[B11] de la Cruz-Ruiz P. Hernando-Rodriguez B. Perez-Jimenez M. M. Rodriguez-Palero M. J. Martinez-Bueno M. D. Pla A. (2021). Prohibitin depletion extends lifespan of a TORC2/SGK-1 mutant through autophagy and the mitochondrial UPR. *Aging Cell* 20:e13359. 10.1111/acel.13359 33939875 PMC8135086

[B12] DePina A. S. Iser W. B. Park S. S. Maudsley S. Wilson M. A. Wolkow C. A. (2011). Regulation of *Caenorhabditis elegans* vitellogenesis by DAF-2/IIS through separable transcriptional and posttranscriptional mechanisms. *BMC Physiol.* 11:11. 10.1186/1472-6793-11-11 21749693 PMC3160409

[B13] Depuydt G. Xie F. Petyuk V. A. Smolders A. Brewer H. M. Camp D. G. II. (2014). LC-MS proteomics analysis of the insulin/IGF-1-deficient *Caenorhabditis elegans* daf-2(e1370) mutant reveals extensive restructuring of intermediary metabolism. *J. Proteome Res.* 13 1938–1956. 10.1021/pr401081b 24555535 PMC3993954

[B14] Diogo C. V. Yambire K. F. Fernandez Mosquera L. Branco F. T. Raimundo N. (2018). Mitochondrial adventures at the organelle society. *Biochem. Biophys. Res. Commun.* 500 87–93. 10.1016/j.bbrc.2017.04.124 28456629 PMC5930832

[B15] Egawa J. Pearn M. L. Lemkuil B. P. Patel P. M. Head B. P. (2016). Membrane lipid rafts and neurobiology: age-related changes in membrane lipids and loss of neuronal function. *J. Physiol.* 594 4565–4579. 10.1113/JP270590 26332795 PMC4983616

[B16] Ezcurra M. Benedetto A. Sornda T. Gilliat A. F. Au C. Zhang Q. (2018). *C. elegans* eats its own intestine to make yolk leading to multiple senescent pathologies. *Curr Biol* 28 2544–2556.e2545. 10.1016/j.cub.2018.06.035 30100339 PMC6108400

[B17] Folch J. Lees M. Sloane Stanley G. H. (1957). A simple method for the isolation and purification of total lipides from animal tissues. *J. Biol. Chem.* 226 497–509. 10.1016/s0021-9258(18)64849-5 13428781

[B18] Gami M. S. Wolkow C. A. (2006). Studies of *Caenorhabditis elegans* DAF-2/insulin signaling reveal targets for pharmacological manipulation of lifespan. *Aging Cell* 5 31–37. 10.1111/j.1474-9726.2006.00188.x 16441841 PMC1413578

[B19] Gao A. W. Smith R. L. van Weeghel M. Kamble R. Janssens G. E. Houtkooper R. H. (2018). Identification of key pathways and metabolic fingerprints of longevity in *C. elegans*. *Exp. Gerontol.* 113 128–140. 10.1016/j.exger.2018.10.003 30300667 PMC6224709

[B20] Gardner B. M. Pincus D. Gotthardt K. Gallagher C. M. Walter P. (2013). Endoplasmic reticulum stress sensing in the unfolded protein response. *Cold Spring Harb, Perspect. Biol.* 5:a013169. 10.1101/cshperspect.a013169 23388626 PMC3578356

[B21] Gatsi R. Schulze B. Rodriguez-Palero M. J. Hernando-Rodriguez B. Baumeister R. Artal-Sanz M. (2014). Prohibitin-mediated lifespan and mitochondrial stress implicate SGK-1, insulin/IGF and mTORC2 in C. elegans. *PLoS One* 9:e107671. 10.1371/journal.pone.0107671 25265021 PMC4180437

[B22] Grant B. Hirsh D. (1999). Receptor-mediated endocytosis in the *Caenorhabditis elegans* oocyte. *Mol. Biol. Cell* 10 4311–4326. 10.1091/mbc.10.12.4311 10588660 PMC25760

[B23] Halaschek-Wiener J. Khattra J. S. McKay S. Pouzyrev A. Stott J. M. Yang G. S. (2005). Analysis of long-lived C. elegans daf-2 mutants using serial analysis of gene expression. *Genome Res.* 15 603–615. 10.1101/gr.3274805 15837805 PMC1088289

[B24] Hall D. H. Hartwieg E. Nguyen K. C. (2012). Modern electron microscopy methods for *C. elegans*. *Methods Cell Biol.* 107 93–149. 10.1016/B978-0-12-394620-1.00004-7 22226522

[B25] Hall D. H. Winfrey V. P. Blaeuer G. Hoffman L. H. Furuta T. Rose K. L. (1999). Ultrastructural features of the adult hermaphrodite gonad of *Caenorhabditis elegans*: relations between the germ line and soma. *Dev. Biol.* 212 101–123. 10.1006/dbio.1999.9356 10419689

[B26] Heine U. Blumenthal T. (1986). Characterization of regions of the *Caenorhabditis elegans* X chromosome containing vitellogenin genes. *J. Mol. Biol.* 188 301–312. 10.1016/0022-2836(86)90156-7 3735423

[B27] Henis-Korenblit S. Zhang P. Hansen M. McCormick M. Lee S. J. Cary M. (2010). Insulin/IGF-1 signaling mutants reprogram ER stress response regulators to promote longevity. *Proc. Natl. Acad. Sci. U.S.A.* 107 9730–9735. 10.1073/pnas.1002575107 20460307 PMC2906894

[B28] Hernando-Rodriguez B. Artal-Sanz M. (2018). Mitochondrial quality control mechanisms and the PHB (Prohibitin) complex. *Cells* 7:268. 10.3390/cells7120238 30501123 PMC6315423

[B29] Hernando-Rodriguez B. Erinjeri A. P. Rodriguez-Palero M. J. Millar V. Gonzalez-Hernandez S. Olmedo M. (2018). Combined flow cytometry and high-throughput image analysis for the study of essential genes in *Caenorhabditis elegans*. *BMC Biol.* 16:36. 10.1186/s12915-018-0496-5 29598825 PMC5875015

[B30] Hou N. S. Gutschmidt A. Choi D. Y. Pather K. Shi X. Watts J. L. (2014). Activation of the endoplasmic reticulum unfolded protein response by lipid disequilibrium without disturbed proteostasis in vivo. *Proc. Natl. Acad. Sci. U.S.A.* 111 E2271–E2280. 10.1073/pnas.1318262111 24843123 PMC4050548

[B31] Hou N. S. Taubert S. (2012). Function and regulation of lipid biology in *Caenorhabditis elegans* Aging. *Front. Physiol.* 3:143. 10.3389/fphys.2012.00143 22629250 PMC3355469

[B32] Jacquemyn J. Cascalho A. Goodchild R. E. (2017). The ins and outs of endoplasmic reticulum-controlled lipid biosynthesis. *EMBO Rep.* 18 1905–1921. 10.15252/embr.201643426 29074503 PMC5666603

[B33] Johnson A. A. Stolzing A. (2019). The role of lipid metabolism in aging, lifespan regulation, and age-related disease. *Aging Cell* 18:e13048. 10.1111/acel.13048 31560163 PMC6826135

[B34] Jove M. Mota-Martorell N. Pradas I. Galo-Licona J. D. Martin-Gari M. Obis E. (2020). The lipidome fingerprint of longevity. *Molecules* 25:4343. 10.3390/molecules25184343 32971886 PMC7570520

[B35] Kenyon C. Chang J. Gensch E. Rudner A. Tabtiang R. (1993). A *C. elegans* mutant that lives twice as long as wild type. *Nature* 366 461–464. 10.1038/366461a0 8247153

[B36] Kenyon C. J. (2010). The genetics of ageing. *Nature* 464 504–512. 10.1038/nature08980 20336132

[B37] Kim S. Sieburth D. (2018). Sphingosine Kinase activates the mitochondrial unfolded protein response and is targeted to mitochondria by stress. *Cell Rep.* 24 2932–2945.e2934. 10.1016/j.celrep.2018.08.037 30208318 PMC6206875

[B38] Kimble J. Sharrock W. J. (1983). Tissue-specific synthesis of yolk proteins in *Caenorhabditis elegans*. *Dev. Biol.* 96 189–196. 10.1016/0012-1606(83)90322-6 6825952

[B39] Kong J. Ji Y. Jeon Y. G. Han J. S. Han K. H. Lee J. H. (2020). Spatiotemporal contact between peroxisomes and lipid droplets regulates fasting-induced lipolysis via PEX5. *Nat. Commun.* 11:578. 10.1038/s41467-019-14176-0 31996685 PMC6989686

[B40] Kornmann B. Currie E. Collins S. R. Schuldiner M. Nunnari J. Weissman J. S. (2009). An ER-mitochondria tethering complex revealed by a synthetic biology screen. *Science* 325 477–481. 10.1126/science.1175088 19556461 PMC2933203

[B41] Lapierre L. R. Gelino S. Melendez A. Hansen M. (2011). Autophagy and lipid metabolism coordinately modulate life span in germline-less *C. elegans*. *Curr. Biol.* 21 1507–1514. 10.1016/j.cub.2011.07.042 21906946 PMC3191188

[B42] Lee J. H. Kong J. Jang J. Y. Han J. S. Ji Y. Lee J. (2014). Lipid droplet protein LID-1 mediates ATGL-1-dependent lipolysis during fasting in *Caenorhabditis elegans*. *Mol. Cell Biol.* 34 4165–4176. 10.1128/MCB.00722-14 25202121 PMC4248714

[B43] Lemieux G. A. Ashrafi K. (2015). Insights and challenges in using *C. elegans* for investigation of fat metabolism. *Crit. Rev. Biochem. Mol. Biol.* 50 69–84. 10.3109/10409238.2014.959890 25228063

[B44] Li W. Kennedy S. G. Ruvkun G. (2003). daf-28 encodes a *C. elegans* insulin superfamily member that is regulated by environmental cues and acts in the DAF-2 signaling pathway. *Genes Dev.* 17 844–858. 10.1101/gad.1066503 12654727 PMC196030

[B45] Liu Y. Samuel B. S. Breen P. C. Ruvkun G. (2014). *Caenorhabditis elegans* pathways that surveil and defend mitochondria. *Nature* 508 406–410. 10.1038/nature13204 24695221 PMC4102179

[B46] Lourenço A. B. Doherty M. K. Whitfield P. D. Artal-Sanz M. (2020). PHB depletion leads to a major remodelling of the lipidome of wild-type and daf-2(e1370) mutant animals at young adult stage. *Mendeley data*

[B47] Lourenco A. B. Munoz-Jimenez C. Venegas-Caleron M. Artal-Sanz M. (2015). Analysis of the effect of the mitochondrial prohibitin complex, a context-dependent modulator of longevity, on the *C. elegans* metabolome. *Biochim. Biophys. Acta* 1847 1457–1468. 10.1016/j.bbabio.2015.06.003 26092086 PMC4580209

[B48] McElwee J. Bubb K. Thomas J. H. (2003). Transcriptional outputs of the *Caenorhabditis elegans* forkhead protein DAF-16. *Aging Cell* 2 111–121. 10.1046/j.1474-9728.2003.00043.x 12882324

[B49] McGhee J. D. (2013). The *Caenorhabditis elegans* intestine. *Wiley Interdiscip. Rev. Dev. Biol.* 2 347–367. 10.1002/wdev.93 23799580

[B50] Munoz-Lobato F. Rodriguez-Palero M. J. Naranjo-Galindo F. J. Shephard F. Gaffney C. J. Szewczyk N. J. (2014). Protective role of DNJ-27/ERdj5 in *Caenorhabditis elegans* models of human neurodegenerative diseases. *Antioxid Redox Signa.l* 20 217–235. 10.1089/ars.2012.5051 23641861 PMC3887457

[B51] Murphy C. T. McCarroll S. A. Bargmann C. I. Fraser A. Kamath R. S. Ahringer J. (2003). Genes that act downstream of DAF-16 to influence the lifespan of *Caenorhabditis elegans*. *Nature* 424 277–283. 10.1038/nature01789 12845331

[B52] Narayan V. Ly T. Pourkarimi E. Murillo A. B. Gartner A. Lamond A. I. (2016). Deep Proteome analysis identifies age-related processes in *C. elegans*. *Cell Syst.* 3 144–159. 10.1016/j.cels.2016.06.011 27453442 PMC5003814

[B53] Nguyen T. B. Louie S. M. Daniele J. R. Tran Q. Dillin A. Zoncu R. (2017). DGAT1-dependent lipid droplet biogenesis protects mitochondrial function during starvation-induced autophagy. *Dev. Cell* 42 9–21.e5. 10.1016/j.devcel.2017.06.003 28697336 PMC5553613

[B54] Niccoli T. Partridge L. (2012). Ageing as a risk factor for disease. *Curr. Biol.* 22 R741–R752. 10.1016/j.cub.2012.07.024 22975005

[B55] Nijtmans L. G. Artal S. M. Grivell L. A. Coates P. J. (2002). The mitochondrial PHB complex: roles in mitochondrial respiratory complex assembly, ageing and degenerative disease. *Cell Mol. Life Sci.* 59 143–155. 10.1007/s00018-002-8411-0 11852914 PMC11337490

[B56] Nijtmans L. G. de Jong L. Artal Sanz M. Coates P. J. Berden J. A. Back J. W. (2000). Prohibitins act as a membrane-bound chaperone for the stabilization of mitochondrial proteins. *EMBO J.* 19 2444–2451. 10.1093/emboj/19.11.2444 10835343 PMC212747

[B57] Ogg S. Paradis S. Gottlieb S. Patterson G. I. Lee L. Tissenbaum H. A. (1997). The Fork head transcription factor DAF-16 transduces insulin-like metabolic and longevity signals in *C. elegans*. *Nature* 389 994–999. 10.1038/40194 9353126

[B58] Olzmann J. A. Carvalho P. (2019). Dynamics and functions of lipid droplets. *Nat. Rev. Mol. Cell Biol.* 20 137–155. 10.1038/s41580-018-0085-z 30523332 PMC6746329

[B59] Osman C. Haag M. Potting C. Rodenfels J. Dip P. V. Wieland F. T. (2009). The genetic interactome of prohibitins: coordinated control of cardiolipin and phosphatidylethanolamine by conserved regulators in mitochondria. *J. Cell Biol.* 184 583–596. 10.1083/jcb.200810189 19221197 PMC2654118

[B60] Palikaras K. Mari M. Petanidou B. Pasparaki A. Filippidis G. Tavernarakis N. (2017). Ectopic fat deposition contributes to age-associated pathology in *Caenorhabditis elegans*. *J. Lipid Res.* 58 72–80. 10.1194/jlr.M069385 27884963 PMC5234711

[B61] Perez C. L. Van Gilst M. R. (2008). A 13C isotope labeling strategy reveals the influence of insulin signaling on lipogenesis in *C. elegans*. *Cell Metab.* 8 266–274. 10.1016/j.cmet.2008.08.007 18762027

[B62] Perez M. F. Lehner B. (2019). Vitellogenins – yolk gene function and regulation in *Caenorhabditis elegans*. *Front. Physiol.* 10:1067. 10.3389/fphys.2019.01067 31551797 PMC6736625

[B63] Phillips M. J. Voeltz G. K. (2016). Structure and function of ER membrane contact sites with other organelles. *Nat. Rev. Mol. Cell Biol.* 17 69–82. 10.1038/nrm.2015.8 26627931 PMC5117888

[B64] Pierce S. B. Costa M. Wisotzkey R. Devadhar S. Homburger S. A. Buchman A. R. (2001). Regulation of DAF-2 receptor signaling by human insulin and ins-1, a member of the unusually large and diverse C. elegans insulin gene family. *Genes Dev.* 15 672–686. 10.1101/gad.867301 11274053 PMC312654

[B65] Prasain J. K. Wilson L. Hoang H. D. Moore R. Miller M. A. (2015). Comparative lipidomics of caenorhabditis elegans metabolic disease models by SWATH non-targeted tandem mass spectrometry. *Metabolites* 5 677–696. 10.3390/metabo5040677 26569325 PMC4693190

[B66] Richter-Dennerlein R. Korwitz A. Haag M. Tatsuta T. Dargazanli S. Baker M. (2014). DNAJC19, a mitochondrial cochaperone associated with cardiomyopathy, forms a complex with prohibitins to regulate cardiolipin remodeling. *Cell Metab.* 20 158–171. 10.1016/j.cmet.2014.04.016 24856930

[B67] Ruzanov P. Riddle D. L. Marra M. A. McKay S. J. Jones S. M. (2007). Genes that may modulate longevity in *C. elegans* in both dauer larvae and long-lived daf-2 adults. *Exp. Gerontol.* 42 825–839. 10.1016/j.exger.2007.04.002 17543485 PMC2755518

[B68] Salas J. J. Martinez-Force E. Garces R. (2006). Phospholipid molecular profiles in the seed kernel from different sunflower (*Helianthus annuus*) mutants. *Lipids* 41 805–811. 10.1007/s11745-006-5034-5 17120935

[B69] Satouchi K. Hirano K. Sakaguchi M. Takehara H. Matsuura F. (1993). Phospholipids from the free-living nematode *Caenorhabditis elegans*. *Lipids* 28 837–840. 8231660 10.1007/BF02536239

[B70] Schleit J. Johnson S. C. Bennett C. F. Simko M. Trongtham N. Castanza A. (2013). Molecular mechanisms underlying genotype-dependent responses to dietary restriction. *Aging Cell* 12 1050–1061. 10.1111/acel.12130 23837470 PMC3838465

[B71] Seah N. E. de Magalhaes Filho C. D. Petrashen A. P. Henderson H. R. Laguer J. Gonzalez J. (2016). Autophagy-mediated longevity is modulated by lipoprotein biogenesis. *Autophagy* 12 261–272. 10.1080/15548627.2015.1127464 26671266 PMC4836030

[B72] Senft D. Ronai Z. A. (2015). UPR, autophagy, and mitochondria crosstalk underlies the ER stress response. *Trends Biochem. Sci.* 40 141–148. 10.1016/j.tibs.2015.01.002 25656104 PMC4340752

[B73] Sharrock W. J. Sutherlin M. E. Leske K. Cheng T. K. Kim T. Y. (1990). Two distinct yolk lipoprotein complexes from *Caenorhabditis elegans*. *J. Biol. Chem.* 265 14422–14431. 10.1016/s0021-9258(18)77319-5 2387862

[B74] Shaye D. D. Greenwald I. (2011). OrthoList: a compendium of *C. elegans* genes with human orthologs. *PLoS One* 6:e20085. 10.1371/journal.pone.0020085 21647448 PMC3102077

[B75] Sornda T. Ezcurra M. Kern C. Galimov E. R. Au C. de la Guardia Y. (2019). Production of YP170 vitellogenins promotes intestinal senescence in *C. elegans*. *J. Gerontol. A Biol. Sci. Med. Sci.* 74 1180–1188. 10.1093/gerona/glz067 30854561 PMC6625598

[B76] Spieth J. Blumenthal T. (1985). The Caenorhabditis elegans vitellogenin gene family includes a gene encoding a distantly related protein. *Mol. Cell Biol.* 5 2495–2501. 10.1128/mcb.5.10.2495-2501.1985 3841791 PMC366982

[B77] Steinbaugh M. J. Narasimhan S. D. Robida-Stubbs S. Moronetti Mazzeo L. E. Dreyfuss J. M. Hourihan J. M. (2015). Lipid-mediated regulation of SKN-1/Nrf in response to germ cell absence. *e*L*ife* 4:e07836. 10.7554/eLife.07836 26196144 PMC4541496

[B78] Sugiura A. Mattie S. Prudent J. McBride H. M. (2017). Newly born peroxisomes are a hybrid of mitochondrial and ER-derived pre-peroxisomes. *Nature* 542 251–254. 10.1038/nature21375 28146471

[B79] Tatsuta T. Scharwey M. Langer T. (2014). Mitochondrial lipid trafficking. *Trends Cell Biol.* 24 44–52. 10.1016/j.tcb.2013.07.011 24001776

[B80] Toth M. J. Tchernof A. (2000). Lipid metabolism in the elderly. *Eur. J. Clin. Nutr.* 54(Suppl 3) S121–S125. 11041083 10.1038/sj.ejcn.1601033

[B81] van Meer G. Voelker D. R. Feigenson G. W. (2008). Membrane lipids: where they are and how they behave. *Nat. Rev. Mol. Cell Biol.* 9 112–124. 10.1038/nrm2330 18216768 PMC2642958

[B82] Vance J. E. (2014). MAM (mitochondria-associated membranes) in mammalian cells: lipids and beyond. *Biochim. Biophys. Acta* 1841 595–609. 10.1016/j.bbalip.2013.11.014 24316057

[B83] Vrablik T. L. Petyuk V. A. Larson E. M. Smith R. D. Watts J. L. (2015). Lipidomic and proteomic analysis of *Caenorhabditis elegans* lipid droplets and identification of ACS-4 as a lipid droplet-associated protein. *Biochim. Biophys. Acta* 1851 1337–1345. 10.1016/j.bbalip.2015.06.004 26121959 PMC4561591

[B84] Wang X. Ding D. Wu L. Jiang T. Wu C. Ge Y. (2021). PHB blocks endoplasmic reticulum stress and apoptosis induced by MPTP/MPP(+) in PD models. *J. Chem. Neuroanat.* 113:101922. 10.1016/j.jchemneu.2021.101922 33581266

[B85] Watts J. L. Ristow M. (2017). Lipid and carbohydrate metabolism in *Caenorhabditis elegans*. *Genetics* 207 413–446. 10.1534/genetics.117.300106 28978773 PMC5629314

[B86] Xu J. Taubert S. (2021). Beyond proteostasis: lipid metabolism as a new player in ER Homeostasis. *Metabolites* 11:52. 10.3390/metabo11010052 33466824 PMC7830277

[B87] Xu N. Zhang S. O. Cole R. A. McKinney S. A. Guo F. Haas J. T. (2012). The FATP1-DGAT2 complex facilitates lipid droplet expansion at the ER-lipid droplet interface. *J. Cell Biol.* 198 895–911. 10.1083/jcb.201201139 22927462 PMC3432760

[B88] Zaarur N. Desevin K. Mackenzie J. Lord A. Grishok A. Kandror K. V. (2019). ATGL-1 mediates the effect of dietary restriction and the insulin/IGF-1 signaling pathway on longevity in *C. elegans*. *Mol. Metab.* 27 75–82. 10.1016/j.molmet.2019.07.001 31311719 PMC6717769

[B89] Zhang S. O. Box A. C. Xu N. Le Men J. Yu J. Guo F. (2010). Genetic and dietary regulation of lipid droplet expansion in *Caenorhabditis elegans*. *Proc. Natl. Acad. Sci. U.S.A.* 107 4640–4645. 10.1073/pnas.0912308107 20176933 PMC2842062

[B90] Zhou K. I. Pincus Z. Slack F. J. (2011). Longevity and stress in *Caenorhabditis elegans*. *Aging (Albany NY.)* 3 733–753. 10.18632/aging.100367 21937765 PMC3184976

