# Supplementary material for: The Mitochondrial PHB Complex Determines Lipid Composition and Interacts With the Endoplasmic Reticulum to Regulate Ageing
Source: Front Physiol. 2021 Jul 1;12:696275. doi: 10.3389/fphys.2021.696275 (PMC8281979; doi:10.3389/fphys.2021.696275)
Supplement: Supplementary file 1 [file Table_1.pdf]

Table S1

TLC GC/FID analysis of TAG Fatty Acid composition from whole worms at the young adult stage. μ corresponds to the average value from at least 2 independent replicates, while δ to the standard deviation and CV to the coefficient of variation (δ/μ ∗ 100) of the content of the different fatty acids. P-values are derived from t-test analysis.

	phb-1(RNAi)			***daf-2;phb-1(RNAi)***			**P-value**
**Fatty Acids**	**μ**	**δ**	**CV (%)**	**μ**	**δ**	**CV (%)**	***phb-1(RNAi)* vs *daf-2;phb-1(RNAi)***
**C14:0**	2.06	-	-	4.70	0.96	20.36	-
**C14:1**	4.06	0.50	12.30	8.12	1.69	20.84	0.08
**C15:1**	0.77	0.10	13.20	2.07	0.91	43.86	0.22
**C16:0**	10.00	2.10	20.98	8.91	0.83	9.35	0.57
**C16:1**	20.48	0.98	4.79	25.25	1.46	5.76	0.05
**C18:0**	8.56	0.41	4.78	3.98	0.50	12.59	0.00
**C18:1n7**	42.23	7.54	17.86	36.49	4.92	13.48	0.48
**C18:2n6c**	2.26	0.07	2.90	3.39	2.34	69.23	0.63
**C20:1**	1.15	0.70	60.62	0.33	0.24	72.99	0.45
**C20:3n6**	2.27	0.32	14.28	1.76	0.29	16.65	0.25
**C22:0**	1.15	0.23	19.87	0.77	0.14	17.86	0.17
**C20:4n3**	1.34	0.68	50.82	1.28	0.42	32.94	0.93
**C20:5n3**	4.69	1.82	38.81	2.97	0.64	21.67	0.33
