# Supplementary material for: The Mitochondrial PHB Complex Determines Lipid Composition and Interacts With the Endoplasmic Reticulum to Regulate Ageing
Source: Front Physiol. 2021 Jul 1;12:696275. doi: 10.3389/fphys.2021.696275 (PMC8281979; doi:10.3389/fphys.2021.696275)
Supplement: Supplementary file 2 [file Table_2.pdf]

Table S2. Lifespan data.

Strain	Median	Max[a]	deaths/total[b]	*P*- value[c] *vs. wild type ctrl* RNAi	*P*- value[c] *vs. daf-2 (e1370) crtl* RNAi
wild type, ctrl RNAi	16	21	91/120		
wild type, dnj-21 RNAi	16	19	85/152	0.2150 (ns)	
daf-2 (e1370), ctrl RNAi	47	58	57/95		
daf-2 (e1370), dnj-21 RNAi	65	97	66/149		< 0.0001 (***)
wild type, ctrl RNAi	19	27	54/96		
wild type, dnj-21 RNAi	19	31	59/147	0.3595 (ns)	
daf-2 (e1370), ctrl RNAi	46	65	98/144		
daf-2 (e1370), dnj-21 RNAi	56	70	42/93		0.0029 (**)
daf-2 (e1370), ctrl RNAi	45	55	36/76		
daf-2 (e1370), dnj-21 RNAi	51	69	80/156		< 0.0001 (***)

[a] day where more than 90% of population is dead.

[b] Confirmed death events divided by the total number of animals included in the assay. Total equals the number of animals that died plus the number of animals that were censored. Censored animals include those that scape from the plate surface, dried out or died from internal bagging (particularly high in *dnj-21* depleted animals).

[c] *P*-values were calculated using the log-rank (Mantel-Cox) test.
