# Supplementary figures and images for: The Mitochondrial PHB Complex Determines Lipid Composition and Interacts With the Endoplasmic Reticulum to Regulate Ageing
Source: Front Physiol. 2021 Jul 1;12:696275. doi: 10.3389/fphys.2021.696275 (PMC8281979; doi:10.3389/fphys.2021.696275)

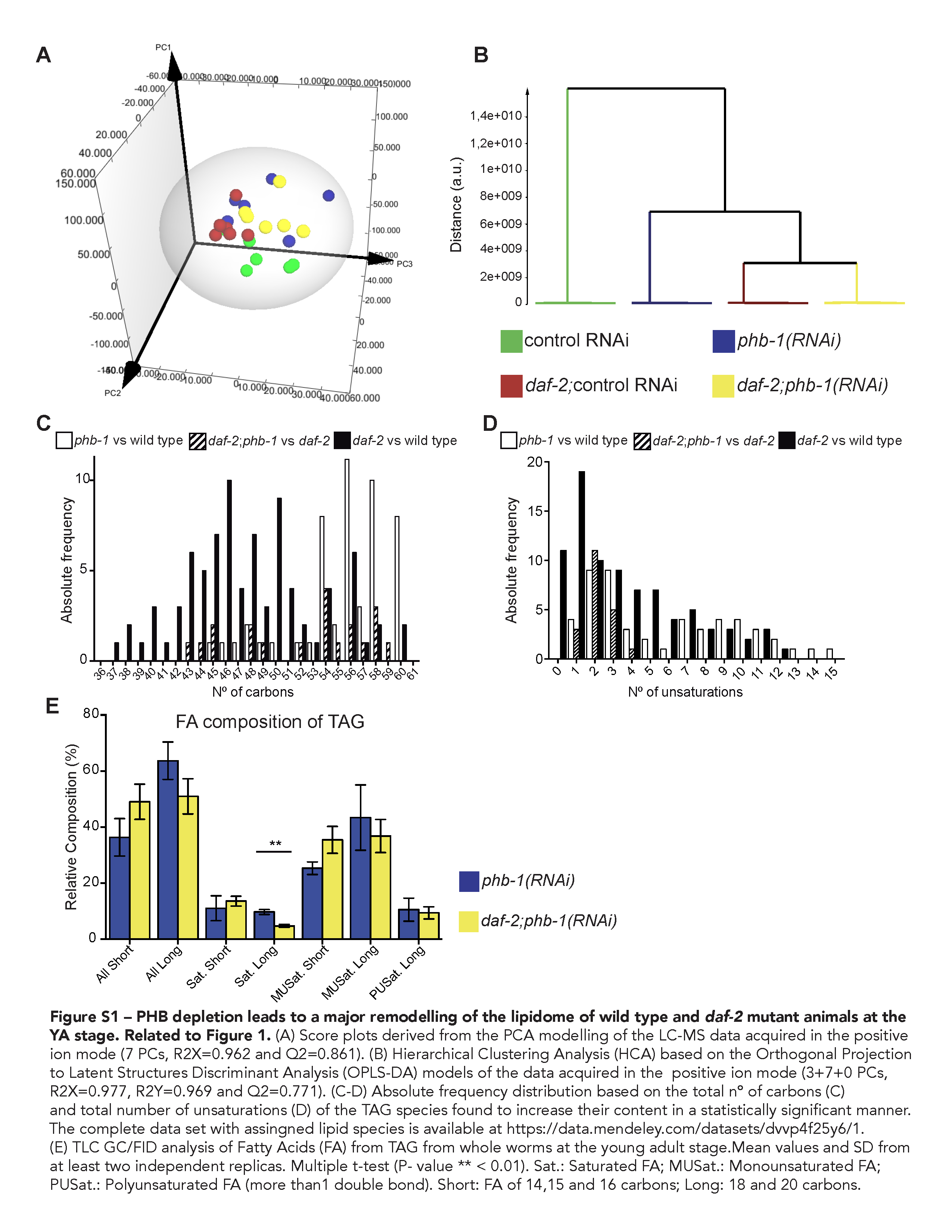

Supplement: Supplementary file 3 [file Image_1.tiff]

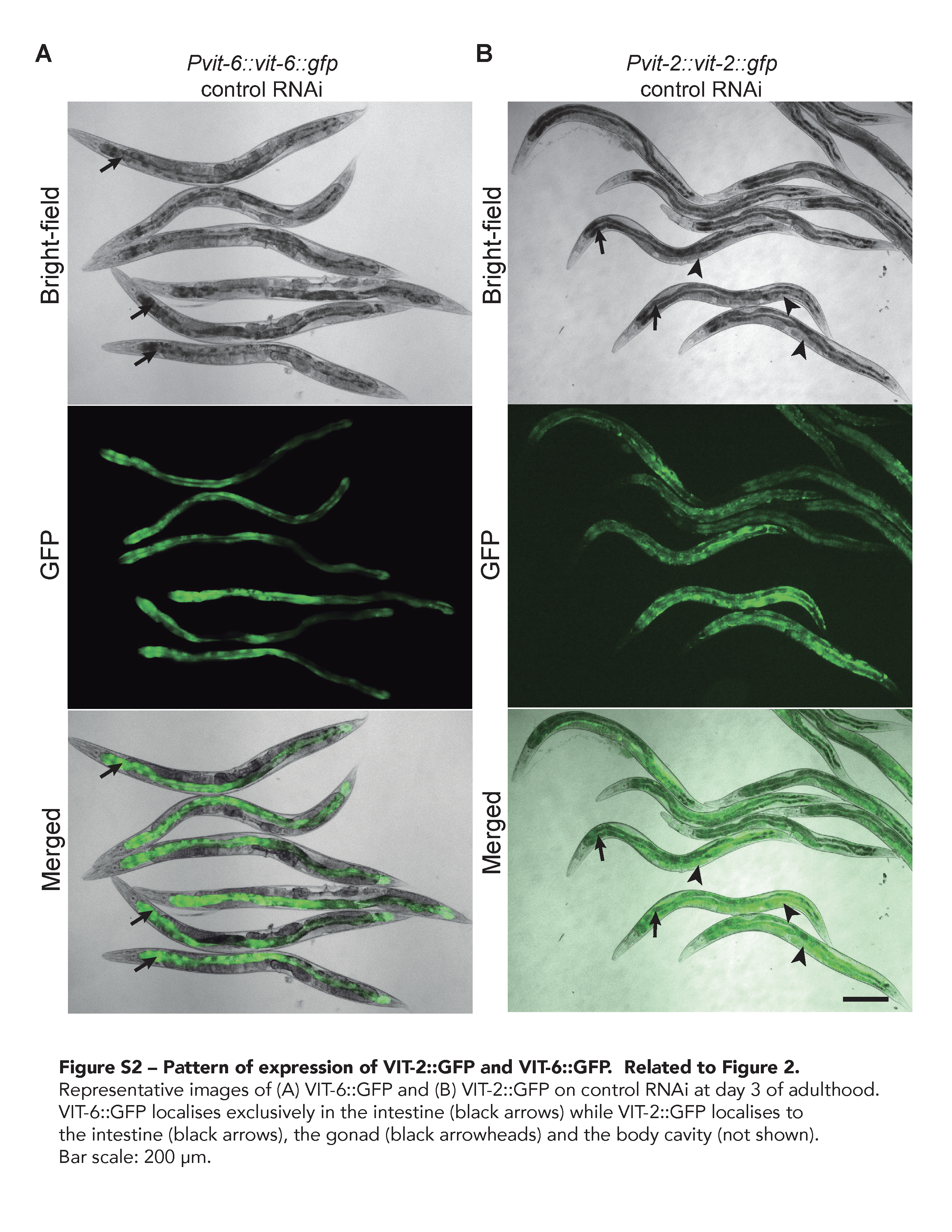

Supplement: Supplementary file 4 [file Image_2.TIFF]

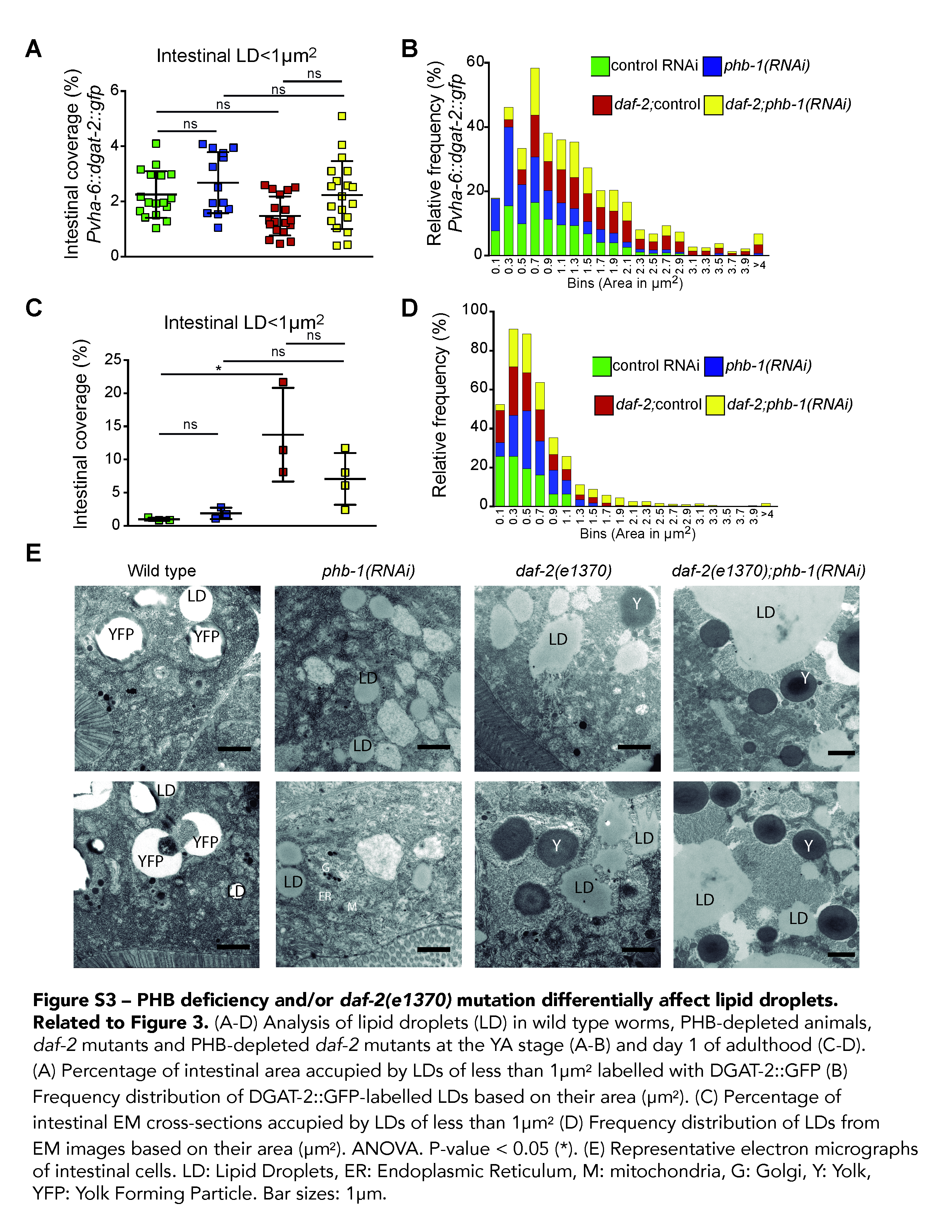

Supplement: Supplementary file 5 [file Image_3.tiff]

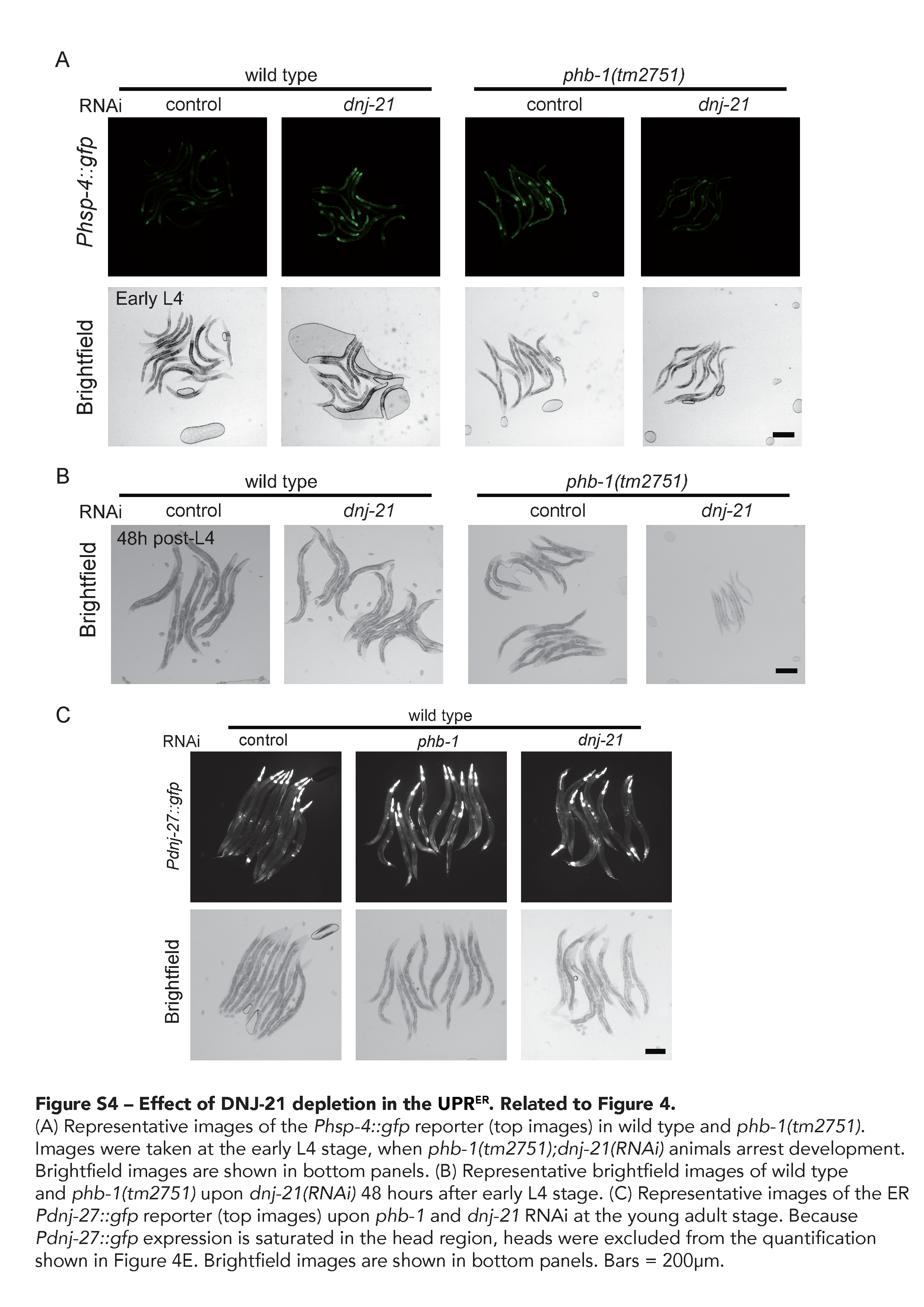

Supplement: Supplementary file 6 [file Image_4.TIFF]
